# Genetic variants and mRNA expression levels of *KLF4* and *KLF5* with hypertension: A combination of case-control study and cohort study

**DOI:** 10.7555/JBR.38.20240208

**Published:** 2024-08-27

**Authors:** Xu Han, Wen Li, Changying Chen, Jiahui Liu, Junxiang Sun, Feifan Wang, Chao Wang, Jialing Mu, Xincheng Gu, Fangyuan Liu, Hankun Xie, Song Yang, Chong Shen

**Affiliations:** 1 Department of Epidemiology, Center for Global Health, School of Public Health, Nanjing Medical University, Nanjing, Jiangsu 211166, China; 2 Department of Cardiology, the Affiliated Yixing People's Hospital of Jiangsu University, People's Hospital of Yixing City, Wuxi, Jiangsu 214200, China; 3 Department of Environmental Genomics, School of Public Health, Nanjing Medical University, Nanjing, Jiangsu 211166, China

**Keywords:** hypertension, *KLF4*, *KLF5*, single-nucleotide polymorphism, mRNA expression, antihypertensive drugs

## Abstract

Hypertension (HT) is a major risk factor for cardiovascular diseases. Krüppel-like factors (KLFs) are important transcription factors in eukaryotes. Studies have reported that KLF4 and KLF5 are correlated with several cardiovascular diseases, but population-based studies on associations between HT and KLF4 or KLF5 have rarely been reported. Therefore, the current study investigated the associations of genetic variants and mRNA expression levels of *KLF4* and *KLF5* with HT, as well as the effects of antihypertensive drugs on the expression levels of these genes. The associations of one single-nucleotide polymorphism (SNP) in *KLF4* and three SNPs in *KLF5* with HT were analyzed using a combination of case-control and cohort studies. The study populations were selected from a community-based cohort in four regions of Jiangsu province. The risks of HT were estimated through logistic and Cox regression analyses. In addition, mRNA expression levels of *KLF4* and *KLF5* were detected in 246 controls and 385 HT cases selected from the aforementioned cohort. Among the HT cases, 263 were not taking antihypertensive drugs [AHD(−)] and 122 were taking antihypertensive drugs [AHD(+)]. In the case-control study, SNP rs9573096 (C>T) in *KLF5* was significantly associated with an increased risk of HT in the additive model (adjusted odds ratio [OR], 1.106; 95% confidence interval [CI], 1.009 to 1.212). In the cohort study of the normotensive population, rs9573096 in *KLF5* was also significantly associated with an increased risk of HT in the additive model (adjusted hazards ratio [HR], 1.199; 95% CI, 1.070 to 1.344). *KLF4* and *KLF5* mRNA expression levels were significantly higher in the AHD(−) group than in the control group (*P* < 0.05), but lower in the AHD(+) group than in the AHD(−) group (*P* < 0.05). The current study demonstrated the associations of *KLF4* and *KLF5* genetic variants with hypertension, as well as the association of the indicative variations in mRNA expression levels of *KLF4* and *KLF5* with the risk of hypertension and antihypertensive treatment.

## Introduction

Hypertension (HT) is a critical risk factor for various health conditions, including ischemic heart disease, stroke, chronic kidney disease, and dementia. The prevalence of HT was approximately 34.0% among 1.508 million individuals surveyed across 92 countries, with a global age- and sex-standardized prevalence of approximately 32.5%^[1]^. HT is a multifactorial disease caused by a complex interaction between genes and environmental conditions, with genetic factors accounting for 20% to 60% of the disease's etiology^[2]^. The main manifestation of HT is a sustained elevation in peripheral resistance and vascular tone^[3]^, while low-grade inflammation plays a role in initiating and maintaining high blood pressure^[4]^. Along with lifestyle changes, medication is the main treatment for HT. Studies have demonstrated that treatment with antihypertensive drugs helps ease the body's chronic inflammatory response^[5–7]^.

Krüppel-like factors (KLFs) are important basic transcription factors in eukaryotes, involved in cell differentiation and proliferation, and other physiological processes^[8]^. Various studies indicate that *KLF4* affects multiple cardiovascular diseases, such as heart failure, myocardial infarction, and dilated cardiomyopathy^[9–11]^. In patients with pulmonary arterial hypertension, the expression levels of *KLF4* were significantly upregulated in small pulmonary artery smooth muscle cells^[12]^. Similarly, during the process of atherosclerosis, the expression levels of *KLF5* were also significantly upregulated in the activated vascular smooth muscle cells (VSMCs)^[13–14]^. Additionally, studies have demonstrated that the mRNA levels of *Klf5* in VSMCs were higher in spontaneously hypertensive rats than in normal rats^[15]^. In mice, KLF5 has been found to play a role in the development of aortic thickening induced by hypertension^[16]^. These findings suggest that KLF4 and KLF5 may be involved in the regulation of pathophysiological processes in cardiovascular diseases. However, population-based studies on the association between HT and *KLF4* or *KLF5* remain limited, and no studies have investigated how antihypertensive drugs affect the mRNA levels of *KLF4* and *KLF5*.

In the current study, we aimed to investigate the associations of genetic variants and mRNA levels of both *KLF4* and *KLF5* with HT in Chinese populations, as well as to determine whether antihypertensive drugs affect blood pressure by altering the expression levels of *KLF4* and *KLF5*.

## Subjects and methods

### Study populations

**Figure 1:**
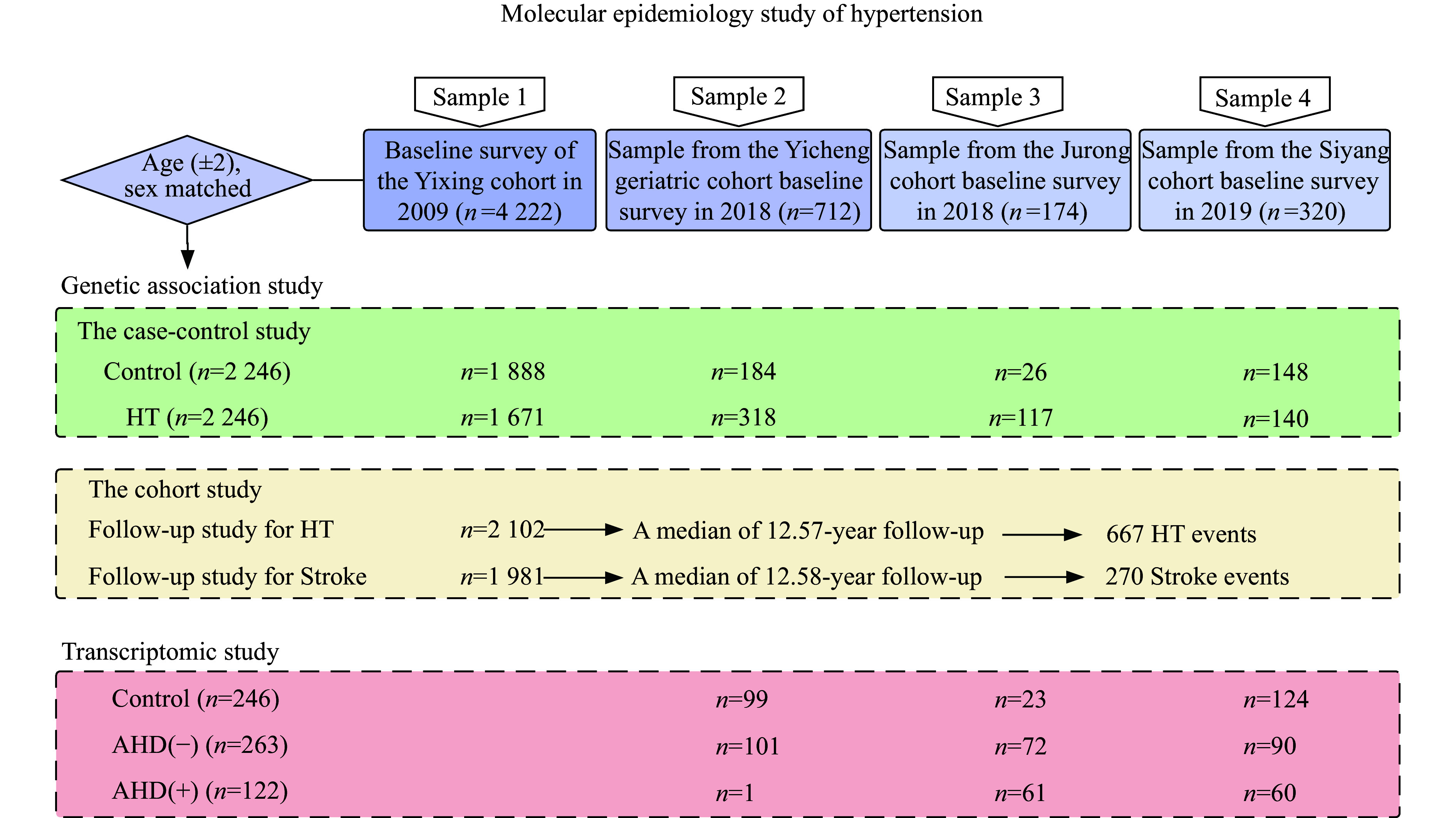
A comprehensive flow chart of the study design. Surveys were conducted in 2009, 2018, and 2019 on community populations in Yixing City, Jurong City, and Siyang City, Jiangsu province, respectively. Abbreviation: HT, hypertension.

We used a combination of case-control and cohort studies to evaluate the risk associations with genetic variants (***Fig. 1***). The study populations were derived from natural population cohorts. Sample 1 was derived from the baseline survey of the Yixing cohort conducted in 2009, which included 4222 participants^[17]^. Samples 2 and 3 were taken from the baseline survey of the Yicheng geriatric cohort and Jurong cohort conducted in 2018, which included 712 and 174 participants, respectively. Sample 4 was taken from the baseline survey of the Siyang cohort conducted in 2019 and contained 320 participants. After being matched by age (± 2) and sex, a total of 4492 subjects were included in the case-control study, which consisted of 2246 HT cases and 2246 controls.

The cohort study of 2102 normotensive subjects and 1981 hypertensive subjects in Yixing City was conducted in 2009. Incident hypertension cases among normotensive subjects and incident stroke cases among hypertensive subjects were collected. As of May 25, 2022, 667 new cases of HT occurred in the normotensive population, and 270 new cases of stroke occurred in the hypertensive population.

In addition, we conducted a transcriptomic study to evaluate the associations between gene expression levels and HT. In total, 246 controls and 385 HT cases were determined with the combined information of hypertension history, medication history, and blood pressure measurements. Among the HT cases, 263 did not receive treatment with antihypertensive drugs [AHD(−)] and 122 received treatment with antihypertensive drugs [AHD(+)].

The information on demographic characteristics, disease history, medication use, and exposure to the primary risk factors in chronic diseases for all the subjects was collected using standardized questionnaires. Additionally, we tracked the incidence of HT and stroke from the local Centers for Disease Control and Prevention (CDC). Smokers were defined as individuals who smoked over 20 cigarettes weekly for a minimum of three months each year. Individuals who had consumed alcohol at least twice a week for six consecutive months or more were classified as alcohol drinkers. Physical examinations included measurements of systolic and diastolic blood pressure (SBP and DBP), which were taken at least three times. All field investigators were trained and certified using the standardized protocols. Hypertension was defined as a self-reported history of the condition, elevated blood pressure (SBP ≥ 140 mmHg, DBP ≥ 90 mmHg), or recent use of antihypertensive medications. Body mass index (BMI) was also calculated. Moreover, peripheral venous blood samples were obtained following an eight-hour fasting period for the measurement of fasting glucose (GLU), triglycerides (TG), high-density lipoprotein cholesterol (HDL-C), total cholesterol (TC), and low-density lipoprotein cholesterol (LDL-C).

The study protocols were approved by the Institutional Review Board of Nanjing Medical University: #200803307 for the Yixing cohort, #2018571 for the Yicheng geriatric cohort, #2015111 for the Jurong cohort, and #2019929 for the Siyang cohort. The written informed consent was obtained from each participant.

### Blood sample collection and leukocyte separation

Under fasting conditions in the morning, 5 mL of venous blood was collected from the study participants and placed in vacuum anticoagulation tubes containing ethylenediaminetetraacetic acid dipotassium salt. The leukocytes were centrifuged in a gradient at 1985 *g* for 5 min, transferred to 2 mL centrifuge tubes, and stored at −20 ℃.

### SNP selection and genotyping

To analyze the regulatory variants, the upstream 2-kb and downstream 1-kb sequences of each candidate gene were included in the analysis. According to the database of the Chinese Han population in Beijing (CHB) and China of the International HapMap Project, SNPs with a minor allele frequency > 0.05 and Hardy-Weinberg equilibrium (HWE) test *P* > 0.05 were screened for tagSNPs by *r*^2^ ≥ 0.8. The bioinformatic functional prediction analysis was performed for all SNPs in each tagSNP subset (https://manticore.niehs.nih.gov/snpinfo/snptag.html/), and sites predicted to be functional were selected based on scores such as exons, promoter start sites, TF binding sites, exonic splicing enhancers, *etc*. ***Supplementary Table 1*** (available online) summarizes the biological forecasts related to these SNPs.

To extract DNA from peripheral venous anticoagulated blood, we used the protein precipitation method (Eaglink, Nanjing, Jiangsu, China). The samples were then stored at −20 ℃. The concentration of DNA samples was normalized and measured using the NanoDrop 2000 spectrophotometer (Thermo Fisher Scientific, Waltham, MA, USA). The polymerase chain reaction (PCR)-TaqMan MGB probe array was performed using the GeneAmp® PCR system 9700 thermal cycler and ABI 7900 system (Applied BioSystems, Foster City, CA, USA). We achieved a 100% successful call rate for all four tagSNPs.

### RNA extraction and reverse transcription

The human peripheral blood RNA protection additive (Eaglink) was used to preserve the rapidly isolated leukocytes. Extraction of total RNA was performed by the RNA extraction kit (Cat. Yu-BR02-1, Yuan, China), and then quantified by NanoDrop 2000 spectrophotometer (Thermo Fisher Scientific). RNA was reversely transcribed to cDNA in the presence of the Reverse Transcription Reagent PrimeScript^TM^ RT Reagent Kit (Cat. RR047A, Takara, Japan). cDNA was stored at −80 ℃ and slowly thawed when removed for detection.

### Examination of *KLF4* and *KLF5* mRNA expression levels

The expression levels of *KLF4* and *KLF5* mRNA in peripheral blood leukocytes were detected by SYBR Green real-time quantitative PCR with three replicate wells set up for each sample, and the experiment was performed in a 384-well plate. The housekeeping gene *GAPDH* was selected as the endogenous control. To analyze mRNA expression levels, the following primer sequences were used: *KLF4* forward primer sequence (5′–3′) CCCACATGAAGCGACTTCCC and reverse primer sequence (5′–3′) CAGGTCCAGGAGATCGTTGAA; *KLF5* forward primer sequence (5′–3′) TCAGTCGTAGACCAGTTCTTCA and reverse primer sequence (5′–3′) CTGGGATTTGTAGAGGCCAGT; and *GAPDH* forward primer sequence (5′–3′) GGAGCGAGATCCCTCCAAAAT and reverse primer sequence (5′–3′) GGCTGTTGTCATACTTCTCATGG. The reaction procedure consisted of initial denaturation at 95 ℃ for 5 min, followed by denaturation at 95 ℃ for 10 s, annealing at 60 ℃ for 20 s, and extension at 72 ℃ for 20 s. To calculate the relative expression levels of *KLFs*, the 2^−ΔΔCT^ method was used as follows^[18]^. Fold change was calculated as the ratio of the median relative gene expression levels between groups. The 2^–ΔΔCT^ method: (1) ΔCT = CT_target gene_ – CT_reference gene_; (2) Coefficient of batch (fn) = mean ΔCT_controls of batch *n*_/mean ΔCT_all controls_ (*n* = batch number); (3) ΔCT_normalized_ = ΔCT/fn; (4) ΔΔCT_normalized_ = ΔCT_normalized_/mean ΔCT_all controls_; (5) Gene expression = 2^−ΔΔCT normalized^.

### Statistical analysis

Continuous variables that were non-normally distributed were expressed as median and inter-quartile range, and the Mann-Whitney test or Kruskal-Wallis test was used to compare differences among groups. Categorical variables were expressed as numbers (percentages), and the Chi-square test (*χ*^*2*^) was used to compare the differences between control and HT groups. Fisher's exact test was used to estimate whether the genotype frequencies of the control or HT group conformed to the HWE law.

The binary logistic regression was applied to calculate odds ratios (ORs) and their corresponding 95% confidence intervals (CIs) for the associations between genetic variants and HT, with adjustment for covariates (*i.e.*, age, sex, smoking, drinking, diabetes, dyslipidemia, and BMI). In the cohort study, the Cox proportional hazards regression was used to estimate hazards ratios (HRs) and 95% CIs. The Jonckheere-Terpstra test was used to evaluate the trend of the expression levels of mRNA across genotypes. The haplotype association was estimated by the Haplo.stats package version 1.9.3 (https://cran.rproject.org/web/packages/haplo.stats/index.html) elsewhere for details^[19–20]^. The Spearman's rank correlation method was used to assess the correlations among mRNA expression levels of *KLFs*, blood pressure, hypersensitive C-reactive protein (hs-CRP), and other quantitative traits. The restricted cubic spline (RCS) regression with four knots was used to model the association curves between mRNA expression levels of *KLFs* and the risk of hypertension.

Several sensitivity analyses were performed to ensure the robustness of the results. In the association analyses of *KLF4* and *KLF5* variants with HT, populations outside of Yixing City were excluded to account for regional differences and validate the results of the case-control study. In addition, subjects with follow-up within one year were excluded to further test the robustness of our results in the cohort study. All data were analyzed using SAS software version 9.4 (SAS Inc., Cary, N.C., USA) and R version 4.2.3. Statistical significance was defined as a two-tailed *P* value < 0.05.

## Results

### Demographic and clinical features of the study population

***Table 1*** presents the detailed demographic and clinical characteristics of the 2246 HT cases and 2246 controls in the case-control study. The median age of the HT group (61.02 years) was comparable with that of the control group (60.33 years, *P* = 0.057). The demographic and clinical characteristics of normotensive and hypertensive subjects in the cohort study are also shown in ***Table 1***. In addition, the expression levels of *KLF4* and *KLF5* mRNA were detected in 246 controls and 385 HT cases, and their demographic information and clinical characteristics are shown in ***Supplementary Table 2*** (available online).

**Table 1:** Demographic and clinical characteristics of the study population in the genetic study

Characteristics	Subjects in the case-control study				Normotensive subjects in the cohort study (*n*=2102)	Hypertensive subjects in the cohort study (*n*=1981)
	Control (*n*=2 246)	HT (*n*=2 246)	*Z/χ* ^*2*^	*P*		
Age (years)^a^	60.33 (53.01, 68.00)	61.02 (54.00, 68.45)	1.903	0.057^b^	57.19 (50.81, 64.01)	61.08 (54.44, 69.46)
Sex [*n* (%)]						
Male	975 (43.4)	975 (43.4)	0.000	1.000^c^	847 (40.3)	807 (40.7)
Female	1271 (56.6)	1271 (56.6)			1255 (59.7)	1174 (59.3)
SBP (mmHg)^a^	128 (120, 134)	145 (138, 155)	43.989	**<0.001** ^**b**^	127 (119, 133)	141 (136, 150)
DBP (mmHg)^a^	80 (77, 83)	88 (80, 93)	29.814	**<0.001** ^**b**^	80 (76, 83)	88 (80, 93)
GLU (mmol/L)^a^	5.23 (4.81, 5.72)	5.42 (4.99, 6.04)	9.496	**<0.001** ^**b**^	5.22 (4.77, 5.68)	5.34 (4.94, 5.94)
TC (mmol/L)^a^	4.78 (4.18, 5.37)	4.86 (4.27, 5.56)	4.176	**<0.001** ^**b**^	4.77 (4.17, 5.37)	4.86 (4.27, 5.57)
TG (mmol/L)^a^	1.20 (0.84, 1.83)	1.45 (0.98, 2.19)	9.706	**<0.001** ^**b**^	1.19 (0.83, 1.83)	1.45 (0.98, 2.22)
HDL-C (mmol/L)^a^	1.32 (1.12, 1.55)	1.30 (1.13, 1.54)	0.483	0.629^b^	1.33 (1.14, 1.55)	1.33 (1.13, 1.55)
LDL-C (mmol/L)^a^	2.63 (2.18, 3.05)	2.73 (2.26, 3.18)	4.716	**<0.001** ^**b**^	2.59 (2.18, 3.05)	2.70 (2.23, 3.19)
BMI (kg/m^2^)^a^	23.52 (21.51, 25.88)	24.91 (22.68, 27.11)	12.377	**<0.001** ^**b**^	23.42 (21.48, 25.84)	24.56 (22.35, 26.99)
Smoking [*n* (%)]						
No	1 715 (76.4)	1 732 (77.1)	0.319	0.572^c^	1 581 (75.2)	1 511 (76.3)
Yes	531 (23.6)	514 (22.9)			521 (24.8)	470 (23.7)
Drinking [*n* (%)]						
No	1 768 (78.7)	17 84 (79.4)	0.303	0.582^c^	1 638 (77.9)	1 566 (79.1)
Yes	478 (21.3)	462 (20.6)			464 (22.1)	415 (20.9)
Diabetes [*n* (%)]						
No	2 016 (89.8)	1 905 (84.8)	24.277	**<0.001** ^**c**^	1 908 (90.8)	1 710 (86.3)
Yes	230 (10.2)	341 (15.2)			194 (9.2)	271 (13.7)
Dyslipidemia [*n* (%)]						
No	984 (43.8)	815 (36.3)	26.169	**<0.001** ^**c**^	927 (44.1)	707 (35.7)
Yes	1 262 (56.2)	1 431 (63.7)			1 175 (55.9)	1 274 (64.3)
^a^Data are presented as median and inter-quartile range.^b^Analyzed with the Mann-Whitney *U* test.^c^Analyzed with the *χ^2^* test.Bold fonts indicate *P*-values < 0.05.Abbreviations: HT, hypertension; SBP, systolic blood pressure; DBP, diastolic blood pressure; GLU, glucose; TC, total cholesterol; TG, triglyceride; HDL-C, high density lipoprotein-cholesterol; LDL-C, low density lipoprotein-cholesterol; BMI, body mass index.						

### Association of *KLF4* and *KLF5* variants with HT in the case-control study

As shown in ***Table 2***, the genotype and allele distributions of rs2236599 in *KLF4* and rs9573096 in *KLF5* followed HWE in both the control and HT groups (both *P*-values > 0.05). The allele frequencies of rs11841945 in *KLF5* were inconsistent with HWE in the control group (*P* = 0.025) but consistent with HWE in the HT group (*P* = 0.454), whereas the allele frequencies of rs3812852 in *KLF5* were inconsistent with HWE in both the control and HT groups (both *P*-values > 0.001).

**Table 2:** Association analyses of *KLF4* and *KLF5* variants with HT in the case-control study

Gene	SNP	Genotype	Groups			OR (95% CI); *P*-value				OR (95% CI); *P*-value^a^		
			Control	HT		Additive model	Dominant model	Recessive model		Additive model	Dominant model	Recessive model
*KLF4*	rs2236599	CC	1 079	1 088								
		CT	959	955								
		TT	208	203		0.985 (0.900–1.078); 0.748	0.984 (0.875–1.106); 0.788	0.974 (0.795–1.193); 0.796		0.994 (0.907–1.090); 0.903	0.992 (0.880–1.118); 0.898	0.995 (0.809–1.224); 0.960
			*P*_HWE_ = 0.808	*P*_HWE_ = 0.751								
*KLF5*	rs11841945	GG	1 388	1 365								
		GC	732	764								
		CC	126	117		1.018 (0.923–1.123); 0.726	1.044 (0.926–1.177); 0.481	0.925 (0.714–1.198); 0.553		1.010 (0.914–1.117); 0.845	1.036 (0.916–1.171); 0.573	0.910 (0.699–1.186); 0.486
			*P*_HWE_ = 0.025	*P*_HWE_ = 0.454								
	rs9573096	CC	1 053	996								
		CT	988	1 006								
		TT	205	244		1.105 (1.010–1.208); **0.029**	1.108 (0.985–1.246); 0.088	1.213 (0.997–1.475); 0.053		1.106 (1.009–1.212); **0.031**	1.116 (0.990–1.259); 0.072	1.193 (0.977–1.458); 0.084
			*P*_HWE_ = 0.217	*P*_HWE_ = 0.673								
	rs3812852	AA	1 956	1 963								
		AG	266	262								
		GG	24	21		0.968 (0.828–1.133); 0.689	0.972 (0.816–1.159); 0.754	0.874 (0.485–1.574); 0.654		0.964 (0.821–1.131); 0.652	0.971 (0.812–1.162); 0.749	0.826 (0.451–1.512); 0.535
			*P*_HWE_ < 0.001	*P*_HWE_ < 0.001								
^a^Adjusted for age, sex, smoking, drinking, diabetes, dyslipidemia, and body mass index.For the additive and the dominant model, the reference genotype is the wild-type homozygote, and for the recessive model, it is the combination of the wild-type homozygote and the heterozygote. Bold fonts indicate *P*-values < 0.05.Abbreviations: OR, odds ratio; CI, confidence interval; HWE, Hardy-Weinberg equilibrium; HT, hypertension.												

In the population of the case-control study, SNP rs9573096 (C>T) in *KLF5* was significantly associated with an increased risk of HT, with an OR (95% CI) of 1.106 (1.009–1.212) in the additive model after adjustment (***Table 2***).

### Association of *KLF4* and *KLF5* variants with risk of HT in the normotensive cohort study

The results of Cox regression analysis showed that SNP rs9573096 (C>T) in *KLF5* was significantly associated with an increased incidence risk of HT in the normotensive subjects of the cohort study, with an HR (95% CI) of 1.199 (1.070–1.344) in the additive model after adjustment (***Table 3***).

**Table 3:** Association analyses of *KLF4* and *KLF5* variants with the incidence risk of HT in the normotensive cohort study

Gene	SNP	Genotype	Total (*n*)	Incidence (*n*)	Person-years	Incidence density/ (10^4^ person-years)	HR (95% CI)				HR (95% CI)^a^		
							Additive model	Dominant model	Recessive model		Additive model	Dominant model	Recessive model
*KLF4*	rs2236599	CC	1002	309	9 750.11	316.92							
		CT	902	294	8 676.54	338.84							
		TT	198	64	1 921.34	333.10	1.047 (0.932–1.175); 0.439	1.069 (0.918–1.245); 0.387	1.031 (0.797–1.334); 0.816		1.054 (0.938–1.183); 0.377	1.081 (0.928–1.260); 0.315	1.034 (0.798–1.338); 0.802
*KLF5*	rs11841945	GG	1308	411	12 700.63	323.61							
		GC	670	212	6 509.49	325.68							
		CC	124	44	1 137.87	386.69	1.047 (0.924–1.187); 0.471	1.026 (0.878–1.199); 0.748	1.206 (0.888–1.638); 0.229		1.039 (0.916–1.178); 0.550	1.018 (0.870–1.190); 0.825	1.185 (0.873–1.609); 0.277
	rs9573096	CC	981	289	9 677.34	298.64							
		CT	921	300	8 817.58	340.23							
		TT	200	78	1 853.07	420.92	1.168 (1.042–1.309); **0.008**	1.184 (1.016–1.380); **0.030**	1.310 (1.035–1.659); **0.025**		1.199 (1.070–1.344); **0.002**	1.224 (1.050–1.427); **0.010**	1.357 (1.071–1.719); **0.012**
	rs3812852	AA	1825	580	17 681.74	328.02							
		AG	249	75	2402.79	312.14							
		GG	28	12	263.46	455.48	1.029 (0.849–1.248); 0.768	1.001 (0.799–1.254); 0.993	1.316 (0.744–2.329); 0.345		1.051 (0.869–1.271); 0.609	1.043 (0.832–1.308); 0.713	1.203 (0.678–2.133); 0.528
^a^Adjusted for age, sex, smoking, drinking, diabetes, dyslipidemia, and body mass index.For the additive and the dominant model, the reference genotype is the wild-type homozygote, and for the recessive model, it is the combination of the wild-type homozygote and the heterozygote. Bold fonts indicate *P*-values < 0.05.Abbreviation: HR, hazards ratio.													

### Association of *KLF4* and *KLF5* variants with risk of stroke among hypertensive subjects in the cohort study

The Cox regression analysis revealed that individuals carrying the CC genotype of rs11841945 in *KLF5* had a higher risk of stroke than those with GG and GC genotypes. After adjustment for potential confounders, the HR was 1.631 (95% CI: 1.021–2.606) (***Supplementary Table 3***, available online).

### Haplotype analysis of rs11841945-rs9573096 with HT

In the case-control study, the haplotype G-T of rs11841945 and rs9573096 in *KLF5* was associated with an increased risk of HT with an adjusted OR (95% CI) of 1.188 (1.051–1.343), compared with the G-C haplotype (***Supplementary Table 4***, available online). Moreover, this G-C haplotype increased the risk of HT by approximately 7.41%, compared with the single locus of rs9573096. This association was further validated in the cohort study of normotensive population, in which the haplotype G-T of rs11841945 and rs9573096 in *KLF5* was associated with an increased risk of developing HT with an adjusted OR (95% CI) of 1.338 (1.106–1.62), compared with the G-C haplotype (***Supplementary Table 4***). Similarly, this G-C haplotype increased the risk of developing HT by approximately 11.59%, compared with the single locus of rs9573096.

### Analysis of blood pressure among genotypes

In the control group of the case-control study, patients with the CC genotype of rs11841945 in *KLF5* had higher SBP (*P* = 0.002) and DBP (*P* = 0.020) than those with the GG and GC genotypes (***Supplementary Fig. 1 A*** and ***1B***, available online). On the contrary, in the AHD(+) group, patients with the CC genotype of rs11841945 in *KLF5* had lower SBP (*P* = 0.031) and DBP (*P* = 0.002) than those with the GG and GC genotypes (***Supplementary Fig. 1 C*** and ***1D***, available online). Detailed results are presented in ***Supplementary Table 5*** (available online).

### The associations of *KLF4* and *KLF5* mRNA expression levels with HT and the effect of antihypertensive drugs on mRNA expression

In the population of the transcriptomic study, the expression levels of *KLF4* mRNA were significantly higher in the AHD(−) group than in the control group (*P* = 0.023), but lower in the AHD(+) group than in the AHD(−) group (*P* = 0.006). In addition, the expression levels of *KLF5* mRNA were significantly lower in the AHD(+) group than in the control group (*P* = 0.034). Detailed results are presented in ***Supplementary Table 6*** (available online).

**Figure 2:**
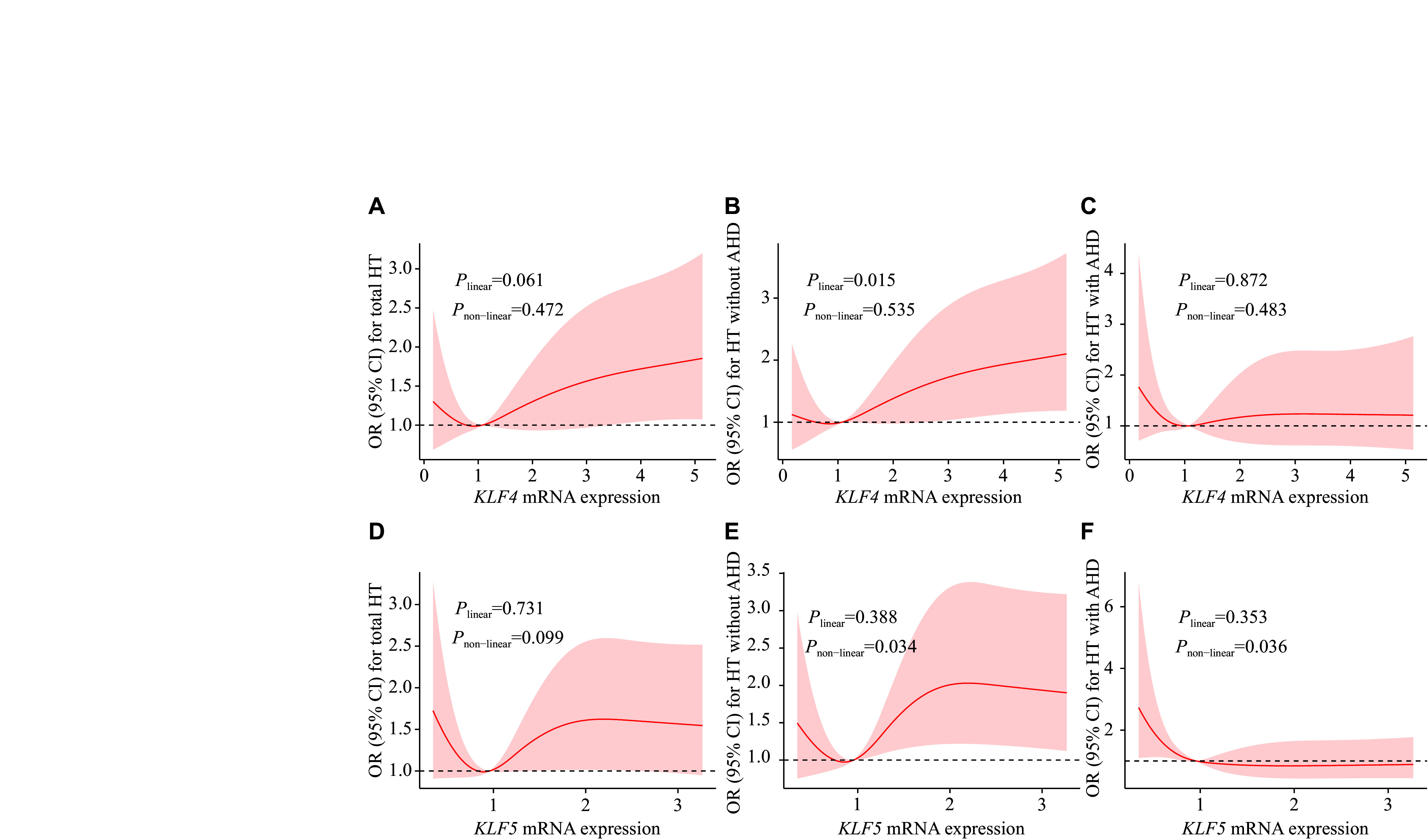
Restricted cubic spline regression analyses for the associations between *KLF* mRNA expression levels and OR of HT. The 95% CIs of the adjusted ORs are represented by the light-red area. The model was adjusted for age, sex, smoking, drinking, diabetes, dyslipidemia, and BMI. Associations of *KLF4* (A–C) and *KLF5* (D–F) mRNA expression levels with OR of HT in three different groups, *i.e.*, total subjects, AHD(−), and AHD(+). Abbreviations: HT, hypertension; OR, odds ratio; CI, confidence interval; BMI, body mass index; AHD(−), subjects not taking antihypertensive drugs; AHD(+), subjects taking antihypertensive drugs.

Further, we performed RCS regression analysis for the associations between *KLFs* mRNA expression levels and the risk of HT (***Fig. 2***). For *KLF4* in the AHD(−)-control group, the RCS regression curve displayed a statistically significant linear trend with HT (*P*_linear_ = 0.015, ***Fig. 2B***). There was a U-shaped association between *KLF5* mRNA levels and OR of HT in the AHD(−)-control comparison group. The OR of HT first decreased and then increased with the increase of *KLF5* mRNA levels, which showed a non-linear relationship (*P*_non-linear_ = 0.034, ***Fig. 2E***). In the AHD(+)-control comparison group, an L-shaped association between *KLF5* mRNA levels and the risk of HT was identified. The OR of HT decreased with the increase of *KLF5* mRNA levels with a non-linear relationship (*P*_non-linear_ = 0.036, ***Fig. 2F***).

No significant difference was observed in *KLF4* and *KLF5* mRNA levels among genotypes (***Supplementary Table 7***, available online).

### Correlation among mRNA expression levels, blood pressure, hs-CRP, and other quantitative traits in the transcriptomic study

After excluding AHD(+), both *KLF4* and *KLF5* mRNA levels were positively correlated with DBP (*ρ* = 0.129, *P* = 0.014 and *ρ* = 0.121, *P* = 0.021, respectively) and mean arterial pressure (*ρ* = 0.130, *P* = 0.014 and *ρ* = 0.116, *P* = 0.028, respectively) in individuals over 55 years old (***Supplementary Table 8***, available online).

There were no significant correlations between hs-CRP levels and blood pressure values (*P* > 0.05), but a negative correlation between *KLF4* mRNA levels and hs-CRP was observed (*ρ* = −0.121, *P* = 0.041; ***Supplementary Table 9***, available online).

### Sensitivity analysis of *KLF4* and *KLF5* polymorphisms with hypertension

In the case-control analysis, the risk for rs9573096 in *KLF5* was slightly increased when other regional populations were excluded, and only the Yixing City population was selected for analysis (***Supplementary Table 10***, available online).

In addition to the above-mentioned analysis, after excluding the participants with less than one year of follow-up, the results for HT/stroke incidence in the cohort study were similar (***Supplementary Table 11*** and ***Supplementary Table 12***, available online). All in all, our conclusions were not substantially altered.

## Discussion

The case-control study demonstrated a significant association between rs9573096 in *KLF5* and an increased risk of HT, which was subsequently validated in the cohort study. The transcriptomic results indicated that the mRNA levels of *KLF4* and *KLF5* were higher in the AHD(−) group than in the AHD(+) group, and that the use of drugs was associated with decreased mRNA levels of *KLF4* and *KLF5*.

The proliferation, migration, and phenotypic conversion of VSMCs are key pathological features of pathological revascularization in HT ^[21–22]^. *KLF4*, a vital transcription factor of VSMCs, plays a significant role in cardiovascular diseases^[23–25]^. Notably, the function of *KLF4* is context-dependent, because it may play opposite roles under different conditions^[26]^. Studies have shown that down-regulating the expression level of *KLF4* effectively inhibited the proliferation and migration of VSMCs^[27–28]^. The expression levels of *KLF4* were low in normal and contractile VSMCs, but increased in response to vascular injury^[29]^. In our findings, the expression levels of *KLF4* mRNA were higher in the AHD(−) group than in the control group, while lower in the AHD(+) group than in the AHD(−) group. RCS regression analysis showed a positive linear correlation between *KLF4* mRNA expression levels and HT in the controls and AHD(−). In general, AHD(+) individuals may have higher blood pressure if they do not receive drugs on time. In our study subjects, the blood pressure of AHD(+) individuals was comparable to that of AHD(−), and the expression levels of *KLF4* mRNA were lower in response to antihypertensive drugs. Therefore, the elevation of *KLF4* mRNA expression levels may lead to an increased risk of HT in the study populations, and antihypertensive drugs may reduce the expression levels of *KLF4*, thereby lowering blood pressure to a certain extent.

In the current study, we identified that the T allele of rs9573096 in *KLF5* was significantly associated with a higher risk of HT in the case-control study, and the association was subsequently validated in the cohort study. These results indicate that the rs9573096 C>T variant in *KLF5* serves as a predisposing marker for the development of HT.

In addition, the SBP (approximately 2 mmHg difference) and DBP (approximately 1 mmHg difference) were higher in the controls with the CC genotype of rs11841945 in *KLF5*. Although these differences were not observed in the HT group, the CC genotype of rs11841945 in *KLF5* was associated with a significantly increased risk of stroke. The haplotype G-T of rs11841945-rs9573096 was identified to be associated with an increased risk of HT in both the case-control and the cohort studies, whereas the C-C haplotype was associated with an increased risk of stroke in the cohort study. These findings explained why the risk associated with the C allele of rs11841945 was not observed in the HT case-control study and the non-HT cohort, while the risk associated with the T allele of rs9573096 was not observed in the cohort study among the subjects with hypertension. However, carriers of the CC genotype rs11841945 in *KLF5* had significantly lower SBP (approximately 6 mmHg difference) and DBP (approximately 6 mmHg difference) in the AHD(+) population. These results indicate that, although carriers of the CC genotype rs11841945 in *KLF5* have a higher risk of elevated blood pressure and stroke, the risk of elevated blood pressure may be significantly reduced with regular drug treatments.

The *KLF5* mRNA levels were elevated in cardiomyocytes from spontaneously hypertensive rats, and hydrogen sulfide reversed this effect^[30]^. Additionally, it has also been demonstrated that expression levels of *KLF5* were significantly higher during differentiation in cells from spontaneously hypertensive rats^[15]^. Similar to *KLF4*, increased levels of *KLF5* mRNA were observed in the AHD(−) group, compared with the control group, while decreased levels of *KLF5* mRNA were observed in the AHD(+) group, compared with both the control and AHD(−) groups in the current study. RCS regression analysis showed a U-shaped association between *KLF5* mRNA levels and HT in the HT subjects not receiving antihypertensive drugs, and an L-shaped association in the HT subjects treated with antihypertensive drugs. These results indicate that *KLF5* is involved in the pathophysiological mechanisms of HT as a transcription factor and that antihypertensive drugs may significantly reduce *KLF5* mRNA expression levels. Under- or over-expression of *KLF5* was associated with HT in the absence of antihypertensive drugs; however, no significant difference in *KLF5* mRNA expression levels was observed among genotypes, indicating the involvement of an unidentified factor. This observation indicates that the polymorphism may not be correlated with alterations in the expression patterns of blood cellular components.

Consequently, we conducted a systematic search of the GTEx database (accessible at https://www.gtexportal.org/) and discovered that, specifically in skin tissues, the rs11841945 and rs9573096 act as splicing quantitative trait loci (sQTLs) for *KLF5*, influencing its splicing patterns. Furthermore, in heart and adipose tissues, the variant rs3812852 functions as an expression quantitative trait locus (eQTL) for *KLF5*, modulating its transcriptional expression levels. Given the multifactorial nature of hypertension, it is imperative to conduct further functional studies to investigate the potential roles of other genetic and non-genetic factors that may contribute to variations in blood pressure.

Since HT is associated with inflammation and *KLF4* and *KLF5* are involved in regulating the body's reaction to inflammatory stress^[26,31]^, we investigated the potential effects of hs-CRP, glucose- and lipid-related markers on disease progression. To better illustrate the correlation between these indicators and the pathogenesis of HT, we created a schematic diagram (***Supplementary Fig. 2***, available online). High levels of serum GLU and TG, along with low levels of HDL-C, were associated with elevated blood pressure. The change in these indices may cause elevated levels of hs-CRP, which triggers an immune-inflammatory response, ultimately leading to higher blood pressure. Individuals with high blood pressure have increased levels of *KLF4* mRNA; however, after taking antihypertensive drugs, the *KLF4* mRNA levels decreased. Despite this, hs-CRP levels remained high, suggesting that while antihypertensive drugs may lower blood pressure by reducing *KLF4* mRNA expression, they do not play an anti-inflammatory role. This observation also suggests that genetic variants and mRNA expression levels of *KLF4* and *KLF5* may regulate blood pressure by targeting specific genes. Protein-protein interaction networks and functional enrichment were analyzed using the String database (https://string-db.org/). Genes such as *CEBPB* interact with *KLF4* and *KLF5* (***Supplementary Fig. 3***, available online) may serve as important target genes for regulating blood pressure^[32]^. Furthermore, it is predicted that *KLF4* and *KLF5* may also be implicated in biological processes such as the STAT family protein binding and miRNA binding^[33]^, which may also play roles in blood pressure regulation.

There are several strengths in the current study. First, we demonstrated that rs9573096 C>T in *KLF5* increased the risk of developing HT in both the case-control and cohort studies. Second, we demonstrated the association of rs11841945 in *KLF5* with blood pressure levels, suggesting that it may be a target for effective drug therapy in hypertensive patients. Finally, the results of differential expression levels of *KLFs* mRNA provided further evidence at the transcriptional level for hypertension pharmacotherapy.

The current study also has some limitations. First, the screening of candidate SNPs with a minor allele frequency > 0.05 did not allow the evaluation of rare variants in *KLF4* and *KLF5* for HT. Second, the effect of various types of antihypertensive drugs on *KLF* expression was not investigated, which limited our ability to assess how different types of antihypertensive drugs affect HT by influencing mRNA expression. Nevertheless, these constraints do not affect the main findings of the current study.

Our findings demonstrate the associations of *KLF4* and *KLF5* genetic variants with hypertension risk, as well as the indicative roles of mRNA expression levels of *KLF4* and *KLF5* in hypertension and antihypertensive treatment. This molecular epidemiological study provides a novel scientific basis for the precise prevention and intervention of HT.

## SUPPLEMENTARY DATA

Supplementary data to this article can be found online.
