# Supplementary material for: Genetic variants and mRNA expression levels of KLF4 and KLF5 with hypertension: A combination of case-control study and cohort study
Source: J Biomed Res. 2024 Aug 27;39(1):103–13. doi: 10.7555/JBR.38.20240208 (PMC11873589; doi:10.7555/JBR.38.20240208)
Supplement: Supplementary file 1 — Supplementary data to this article can be found online. [file jbr-39-1-103-S1.pdf]

Available online at www.jbr-pub.org.cn

Open Access at PubMed Central

Journal of Biomedical Research, 2025 39(1): 1–11

Supplementary Data

JBR

Genetic variants and mRNA expression levels of *KLF4* and *KLF5* with hypertension: A combination of case-control study and cohort study

Xu Han[1,△], Wen Li[1,△], Changying Chen[1,△], Jiahui Liu[1], Junxiang Sun[2], Feifan Wang[1], Chao Wang[3], Jialing Mu[1], Xincheng Gu[1], Fangyuan Liu[1], Hankun Xie[1], Song Yang[2,✉], Chong Shen[1,✉]

[1]*Department of Epidemiology, Center for Global Health, School of Public Health, Nanjing Medical University, Nanjing, Jiangsu 211166, China;*
[2]*Department of Cardiology, the Affiliated Yixing People's Hospital of Jiangsu University, People's Hospital of Yixing City, Wuxi, Jiangsu 214200, China;*
[3]*Department of Environmental Genomics, School of Public Health, Nanjing Medical University, Nanjing, Jiangsu 211166, China.*

Supplementary Table 1 **Biological information and function predictions for the selected tagSNPs**

Nearby gene	SNP	Chromosome (GRCh38.p13)	Allele	Consequence	Enhancer	TFBS	eQTL	MAF (CHB)
KLF4	rs2236599	chr9:107487224	C>T	Synonymous variant	29	3	–	0.274
	rs11841945	chr13:73055921	G>C	Intron variant	13	3	–	0.178
KLF5	rs9573096	chr13:73075195	C>T	Intron variant	25	2	–	0.333
	rs3812852	chr13:73057735	A>G	Intron variant	8	2	–	0.065

Abbreviations: SNP, single nucleotide polymorphism; TFBS, transcription factor binding site; eQTL, expression quantitative trait loci; MAF, minor allele frequency.

Supplementary Table 2 **Demographic and clinical characteristics of subjects in the transcriptomic study**

Characteristics	Group	Control	HT						
			Total		AHD(−)		AHD(+)		
		n=246	*n*=385	*P*[a]	*n*=263	*P*[a]	*n*=122	*P*[a]	*P*[b]
Age (years)[c]		66.00 (53.00, 71.00)	67.00 (56.00, 72.00)	**0.039**[d]	67.00 (55.00,72.00)	0.106[d]	67.00 (58.00,73.00)	**0.043**[d]	0.445[d]
Sex [*n* (%)]	Male	98 (39.8)	172 (44.7)	0.265[e]	112 (42.6)	0.590[e]	60 (49.2)	0.111[e]	0.271[e]
	Female	148 (60.2)	213 (55.3)		151 (57.4)		62 (50.8)		
SBP (mmHg)[c]		126 (118, 131)	152 (144, 163)	**<0.001**[d]	152 (144, 163)	**<0.001**[d]	153 (142, 164)	**<0.001**[d]	0.683[d]
DBP (mmHg)[c]		75 (69, 80)	86 (78, 93)	**<0.001**[d]	86 (77, 93)	**<0.001**[d]	85 (79, 94)	**<0.001**[d]	0.538[d]
GLU (mmol/L)[c]		5.38 (5.04, 5.99)	5.70 (5.23, 6.43)	**<0.001**[d]	5.61 (5.20, 6.06)	**0.005**[d]	6.155 (5.41, 7.77)	**<0.001**[d]	**<0.001**[d]

△These authors contributed equally to this work.

✉Corresponding authors: Chong Shen, Department of Epidemiology, Center for Global Health, School of Public Health, Nanjing Medical University, Longmian Avenue, Jiangning District, Nanjing, Jiangsu 211166, China. E-mail: sc@njmu.edu.cn; Song Yang, Department of Cardiology, the Affiliated Yixing People's Hospital of Jiangsu University, People's Hospital of Yixing City, 75 Tongzhenguan Road, Wuxi, Jiangsu 214200, China. E-mail: staff052@yxph.com.

Received: 11 July 2024; Revised: 09 August 2024; Accepted: 23 August 2024; Published online: 27 August 2024

CLC number: R544.1, Document code: A

The authors reported no conflict of interests.

This is an open access article under the Creative Commons Attribution (CC BY 4.0) license, which permits others to distribute, remix, adapt and build upon this work, for commercial use, provided the original work is properly cited.

© **2025 by Journal of Biomedical Research.**

https://doi.org/10.7555/JBR.38.20240208

Supplementary Table 2 **Demographic and clinical characteristics of subjects in the transcriptomic study** (continued)

Characteristics	Group	Control	HT						
			Total		AHD(−)		AHD(+)		
		n=246	*n*=385	P^a	*n*=263	P^a	*n*=122	P^a	P^b
TC (mmol/L)[c]		4.68 (3.97, 5.25)	4.82 (4.20, 5.47)	**0.028**[d]	4.86 (4.21, 5.47)	**0.016**[d]	4.72 (4.11, 5.46)	0.356[d]	0.293[d]
TG (mmol/L)[c]		1.15 (0.82, 1.64)	1.40 (0.98, 1.87)	**<0.001**[d]	1.31 (0.96, 1.79)	**0.005**[d]	1.58 (1.10, 2.22)	**<0.001**[d]	**0.003**[d]
HDL-C (mmol/L)[c]		1.28 (1.04, 1.54)	1.29 (1.08, 1.55)	0.411[d]	1.31 (1.10, 1.58)	0.096[d]	1.235 (1.02, 1.47)	0.336[d]	**0.017**[d]
LDL-C (mmol/L)[c]		2.66 (2.04, 3.08)	2.70 (2.17, 3.22)	0.107[d]	2.73 (2.21, 3.24)	0.054[d]	2.61 (2.11, 3.06)	0.682[d]	0.260[d]
BMI (kg/m^2)[c]		23.98 (22.14, 26.29)	25.39 (23.12, 27.59)	**<0.001**[d]	25.15 (22.77, 26.98)	**0.006**[d]	26.40 (23.96, 28.60)	**<0.001**[d]	**<0.001**[d]
Smoking [*n* (%)]	No	213 (86.6)	289 (75.1)	**<0.001**[e]	204 (77.6)	**0.012**[e]	85 (69.7)	**<0.001**[e]	0.124[e]
	Yes	33 (13.4)	96 (24.9)		59 (22.4)		37 (30.3)		
Drinking [*n* (%)]	No	214 (87.0)	294 (76.4)	**<0.001**[e]	209 (79.5)	**0.032**[e]	85 (69.7)	**<0.001**[e]	**0.048**[e]
	Yes	32 (13.0)	91 (23.6)		54 (20.5)		37 (30.3)		
Diabetes [*n* (%)]	No	210 (85.4)	290 (75.3)	**0.003**[e]	222 (84.4)	0.860[e]	68 (55.7)	**<0.001**[e]	**<0.001**[e]
	Yes	36 (14.6)	95 (24.7)		41 (15.6)		54 (44.3)		
Dyslipidemia [*n* (%)]	No	114 (46.3)	155 (40.3)	0.154[e]	115 (43.7)	0.615[e]	40 (32.8)	**0.018**[e]	0.054[e]
	Yes	132 (53.7)	230 (59.7)		148 (56.3)		82 (67.2)		

[a]Compared with the control group.
[b]Compared with the AHD(−).
[c]Data are presented as median and inter-quartile range.
[d]Analyzed with the Mann-Whitney *U* test.
[e]Analyzed with the χ^2 test.
Abbreviations: HT, hypertension; AHD, antihypertensive drug; SBP, systolic blood pressure; DBP, diastolic blood pressure; GLU, glucose; TC, total cholesterol; TG, triglyceride; HDL-C, high-density lipoprotein-cholesterol; LDL-C, low-density lipoprotein-cholesterol; BMI, body mass index.

Supplementary Table 3 **Association analyses of *KLF4* and *KLF5* variants with the incidence risk of stroke in the hypertensive cohort study**

Gene	SNP	Genotype	Total (*n*)	Incidence (*n*)	Person-years	Incidence density/ (10^4 person-years)	HR (95% CI); *P*-value			HR (95% CI); *P*-value[a]		
							Additive model	Dominant model	Recessive model	Additive model	Dominant model	Recessive model
KLF4	rs2236599	CC	981	131	10 812.99	121.15						
		CT	833	112	9 246.83	121.12						
		TT	194	27	2 098.27	128.68	1.021 (0.852–1.225); 0.821	1.013 (0.798–1.286); 0.917	1.068 (0.718–1.590); 0.744	0.993 (0.828–1.192); 0.941	0.979 (0.770–1.244); 0.861	1.027 (0.690–1.529); 0.896
	rs11841945	GG	1 207	161	13 330.86	120.77						
		GC	700	90	7 754.82	116.06						
		CC	101	19	1 072.41	177.17	1.082 (0.886–1.320); 0.440	1.023 (0.802–1.304); 0.855	1.494 (0.937–2.381); 0.092	1.124 (0.920–1.375); 0.253	1.062 (0.832–1.356); 0.631	1.631 (1.021–2.606); **0.041**
KLF5	rs9573096	CC	917	127	10 098.92	125.76						
		CT	884	118	9 816.56	120.20						
		TT	207	25	2 242.60	111.48	0.946 (0.788–1.137); 0.555	0.941 (0.741–1.196); 0.621	0.906 (0.600–1.368); 0.639	0.939 (0.783–1.127); 0.501	0.953 (0.750–1.212); 0.695	0.842 (0.558–1.272); 0.415
	rs3812852	AA	1 741	228	19 267.87	118.33						
		AG	248	40	2 680.47	149.23						
		GG	19	2	209.75	95.35	1.168 (0.868–1.571); 0.305	1.231 (0.886–1.711); 0.215	0.776 (0.193–3.119); 0.721	1.196 (0.880–1.626); 0.253	1.212 (0.870–1.687); 0.256	1.299 (0.321–5.262); 0.714

[a]Adjusted for age, sex, smoking, drinking, diabetes, dyslipidemia, and BMI. Abbreviation: HR, hazards ratio.

Supplementary Table 4 **Haplotype frequencies of rs11841945-rs9573096 and the association in different studies**

Study design	Haplotype[a]	Total	Case	Control	OR (95% CI)	*P*	OR (95% CI)[c]	*P*[c]
Case-control study	G-C[b]	0.600	0.588	0.611	Reference		Reference	
Total (*n*=4 492)	G-T	0.180	0.190	0.170	1.169 (1.037–1.317)	**0.018**	1.188 (1.051–1.343)	**0.010**
HT cases (*n*=2 246)	C-C	0.078	0.079	0.077	1.068 (0.896–1.274)	0.995	1.085 (0.906–1.299)	0.934
Control (*n*=2 246)	C-T	0.180	0.190	0.170	1.051 (0.928–1.189)	0.659	1.036 (0.913–1.176)	0.854
Cohort study of HT incidence	G-C[b]	0.608	0.580	0.621	Reference		Reference	
Total (*n*=2 102)	G-T	0.173	0.195	0.164	1.283 (1.065–1.546)	**0.015**	1.338 (1.106–1.620)	**0.005**
HT incidence (*n*=667)	C-C	0.078	0.078	0.077	1.071 (0.816–1.407)	0.830	1.070 (0.809–1.416)	0.731
	C-T	0.141	0.147	0.138	1.144 (0.943–1.387)	0.312	1.149 (0.943–1.401)	0.326
Cohort study of stroke incidence	G-C[b]	0.595	0.579	0.597	Reference		Reference	
Total (*n*=1 981)	G-T	0.182	0.184	0.181	1.045 (0.800–1.364)	0.802	1.022 (0.778–1.342)	0.603
Stroke incidence (*n*=270)	C-C	0.083	0.110	0.079	1.423 (1.011–2.004)	**0.040**	1.529 (1.070–2.185)	**0.015**
	C-T	0.141	0.127	0.143	0.928 (0.695–1.239)	0.573	0.953 (0.707–1.284)	0.711

[a]Loci are arranged in the order rs11841945-rs9573096.
[b]G-C was chosen to be the reference.
[c]Adjusted for age, sex, smoking, drinking, diabetes, dyslipidemia, and body mass index.
Abbreviations: HT, hypertension; OR, odds ratio; CI, confidence interval.

Supplementary Table 5 **Quantitative trait analysis for blood pressure between genotypes**

Gene	SNPs	Genotype	Control			AHD(−)			AHD(+)		
			n	SBP (mmHg)[a]	DBP (mmHg)[a]	*n*	SBP (mmHg)[a]	DBP (mmHg)[a]	*n*	SBP (mmHg)[a]	DBP (mmHg)[a]
KLF5	rs11841945	GG+GC	2 120	128 (120, 134)	80 (77, 83)	1 081	145 (140, 154)	88 (80, 93)	1 048	144 (135, 155)	88 (80, 94)
		CC	126	130 (123, 136)	80 (78, 85)	63	143 (140, 149)	88 (80, 93)	54	138 (130, 150)	82 (78, 90)
		Z		3.1387	2.325		1.127	0.329		2.157	3.069
		P[b]		**0.002**	**0.020**		0.260	0.743		**0.031**	**0.002**
	rs9573096	CC	1 053	128 (120, 134)	80 (77, 83)	511	144 (139, 151)	88 (80, 93)[a]	485	143 (133, 155)	88 (80, 94)
		CT+TT	1 193	127 (120, 133)	80 (77, 83)	633	145 (140, 154)	88 (81, 93)[a]	617	144 (135, 155)	87 (80, 94)
		Z		1.1266	0.1576		2.465	0.2184		0.932	0.563
		P[b]		0.260	0.875		**0.014**	0.827		0.352	0.573

[a]Data are presented as median and inter-quartile range.
[b]Mann-Whitney *U* test.

Supplementary Table 6 **Differential analyses of *KLFs* mRNA expression levels among groups**

mRNA			Control		HT cases														
					Total				AHD(−)				AHD(+)						
			n	mRNA expression ($2^{-\Delta\Delta CT}$)[a]	*n*	mRNA expression ($2^{-\Delta\Delta CT}$)[a]	FC[b]	*P*[b]	*n*	mRNA expression ($2^{-\Delta\Delta CT}$)[a]	FC[b]	*P*[b]	*n*	mRNA expression ($2^{-\Delta\Delta CT}$)	FC[b]	*P*[b]	FC[c]	*P*[c]
KLF4		Total	246	1.081 (0.637, 1.789)	385	1.199 (0.686, 2.070)	1.109	0.230	263	1.267 (0.719, 2.361)	1.172	**0.023**	122	0.997 (0.602, 1.636)	0.922	0.254	0.787	**0.006**
	Age (years)	≤55	78	1.123 (0.548, 1.983)	95	1.120 (0.647, 1.901)	0.997	0.943	68	1.118 (0.633, 1.991)	0.996	0.948	27	1.120 (0.652, 1.801)	0.997	0.962	1.002	0.951
		>55	168	1.078 (0.662, 1.781)	290	1.237 (0.692, 2.182)	1.147	0.155	195	1.342 (0.823, 2.441)	1.245	**0.007**	95	0.951 (0.579, 1.636)	0.882	0.204	0.709	**0.002**
	Sex	Male	98	1.118 (0.599, 1.983)	172	1.236 (0.631, 2.135)	1.106	0.521	112	1.316 (0.66, 2.444)	1.177	0.221	60	1.067 (0.620, 1.631)	0.954	0.610	0.811	0.118
		Female	148	1.070 (0.666, 1.763)	213	1.125 (0.701, 1.993)	1.051	0.332	151	1.156 (0.764, 2.257)	1.080	0.061	62	0.965 (0.546, 1.801)	0.902	0.247	0.835	**0.018**
	Smoking	No	213	1.067 (0.628, 1.839)	289	1.140 (0.704, 1.993)	1.068	0.220	204	1.254 (0.779, 2.280)	1.175	**0.023**	85	0.967 (0.606, 1.621)	0.906	0.207	0.771	**0.006**
		Yes	33	1.243 (0.852, 1.688)	96	1.253 (0.606, 2.277)	1.008	0.933	59	1.267 (0.629, 2.441)	1.019	0.679	37	1.109 (0.593, 1.932)	0.892	0.682	0.875	0.462
	Drinking	No	214	1.082 (0.676, 1.839)	294	1.215 (0.688, 2.101)	1.123	0.369	209	1.234 (0.719, 2.361)	1.140	0.099	85	1.108 (0.606, 1.817)	1.024	0.360	0.898	**0.048**
		Yes	32	0.902 (0.422, 1.466)	91	1.183 (0.684, 2.005)	1.312	0.153	54	1.316 (0.798, 2.239)	1.459	**0.045**	37	0.928 (0.593, 1.444)	1.029	0.797	0.705	**0.048**
KLF5		Total	246	1.012 (0.770, 1.433)	385	1.033 (0.756, 1.474)	1.021	0.525	263	1.128 (0.802, 1.533)	1.115	**0.037**	122	0.879 (0.619, 1.271)	0.869	**0.034**	0.779	**<0.001**
	Age (years)	≤55	78	1.061 (0.784, 1.446)	95	0.966 (0.746, 1.552)	0.910	0.839	68	0.978 (0.745, 1.534)	0.922	0.804	27	0.943 (0.756, 1.909)	0.889	0.985	0.964	0.944
		>55	168	0.981 (0.767, 1.422)	290	1.067 (0.758, 1.441)	1.088	0.466	195	1.179 (0.815, 1.533)	1.202	**0.018**	95	0.833 (0.603, 1.241)	0.849	**0.022**	0.707	**<0.001**
	Sex	Male	98	1.027 (0.774, 1.409)	172	1.038 (0.796, 1.480)	1.011	0.451	112	1.123 (0.853, 1.480)	1.093	0.141	60	0.911 (0.690, 1.447)	0.887	0.514	0.811	0.087
		Female	148	0.995 (0.769, 1.445)	213	1.031 (0.723, 1.451)	1.036	0.913	151	1.140 (0.788, 1.566)	1.146	0.139	62	0.792 (0.608, 1.212)	0.796	**0.013**	0.695	**0.001**
	Smoking	No	213	0.990 (0.759, 1.446)	289	1.045 (0.728, 1.457)	1.056	0.606	204	1.134 (0.794, 1.528)	1.145	0.090	85	0.884 (0.626, 1.241)	0.893	0.061	0.780	**0.003**
		Yes	33	1.043 (0.865, 1.281)	96	1.012 (0.794, 1.485)	0.970	0.844	59	1.117 (0.884, 1.533)	1.071	0.251	37	0.874 (0.603, 1.329)	0.838	0.221	0.782	0.061
	Drinking	No	214	0.977 (0.764, 1.439)	294	1.043 (0.714, 1.440)	1.068	0.650	209	1.128 (0.794, 1.515)	1.155	0.088	85	0.811 (0.603, 1.241)	0.830	**0.038**	0.719	**0.002**
		Yes	32	1.099 (0.898, 1.406)	91	1.010 (0.848, 1.533)	0.919	0.984	54	1.161 (0.882, 1.533)	1.056	0.372	37	0.891 (0.783, 1.361)	0.811	0.213	0.767	0.077

[a] Data are presented as median and inter-quartile range.
[b] Mann-Whitney *U* test, compared with the Control.
[c] Mann-Whitney *U* test, compared with the AHD(−).
Abbreviations: AHD, antihypertensive drug; FC, fold change.

Supplementary Table 7 **The mRNA expression levels ($2^{-\Delta\Delta CT}$) of *KLF4* and *KLF5* among different genotypes**

Gene	SNPs	Genotype	*n*	Control	*n*	AHD(−)	*n*	AHD(+)
KLF4	rs2236599	CC	130	1.037 (0.628, 1.770)	123	1.218 (0.697, 2.305)	60	1.029 (0.612, 1.664)
		CT	94	1.090 (0.676, 1.756)	118	1.293 (0.822, 2.239)	57	1.012 (0.562, 1.713)
		TT	22	1.730 (0.604, 2.073)	22	1.777 (0.824, 2.474)	5	0.754 (0.747, 1.444)
		H		1.339		1.627		0.151
		P^a		0.512		0.443		0.927
		P_{trend}		0.386		0.221		0.878
KLF5	rs11841945	GG	159	1.029 (0.759, 1.451)	168	1.134 (0.801, 1.549)	71	0.848 (0.619, 1.220)
		GC	77	0.985 (0.806, 1.385)	79	1.114 (0.777, 1.515)	46	0.927 (0.626, 1.289)
		CC	10	0.862 (0.700, 1.055)	16	1.318 (0.926, 1.844)	5	0.833 (0.427, 0.915)
		H		1.701		1.192		1.434
		P^a		0.427		0.551		0.488
		P_{trend}		0.641		0.818		0.757
	rs9573096	CC	115	1.029 (0.792, 1.430)	118	1.080 (0.706, 1.514)	59	0.819 (0.603, 1.260)
		CT	111	1.005 (0.705, 1.481)	116	1.179 (0.831, 1.499)	51	0.937 (0.756, 1.299)
		TT	20	0.919 (0.759, 1.236)	29	1.288 (1.006, 1.703)	12	0.784 (0.543, 0.943)
		H		0.429		3.021		3.113
		P^a		0.807		0.221		0.211
		P_{trend}		0.676		0.095		0.785
	rs3812852	AA	208	1.027 (0.784, 1.445)	233	–	111	0.873 (0.603, 1.271)
		AG	36	0.939 (0.580, 1.274)	30	–	9	0.937 (0.792, 1.225)
		GG	2	1.093 (0.774, 1.411)	0	–	2	1.066 (0.771, 1.361)
		H		1.605		–		0.332
		P^a		0.448		–		0.847
		P_{trend}		0.220		–		0.577

mRNA levels are presented as median and inter-quartile range.
[a]Kruskal-Wallis test was performed.

***Supplementary Table 8* Correlation between *KLFs* mRNA expression and blood pressure[a]**

Group			Total								Participants w/o AHD(+)								AHD(+)							
			KLF4				*KLF5*				*KLF4*				*KLF5*				*KLF4*				*KLF5*			
			SBP	DBP	PP	MAP	SBP	DBP	PP	MAP	SBP	DBP	PP	MAP	SBP	DBP	PP	MAP	SBP	DBP	PP	MAP	SBP	DBP	PP	MAP
Total		ρ	0.029	0.033	0.028	0.043	0.005	0.025	0.001	0.024	0.054	0.051	0.039	0.069	0.048	0.074	0.017	0.077	0.031	0.032	0.031	0.036	0.049	−0.038	0.075	−0.002
		P	0.470	0.402	0.476	0.285	0.905	0.536	0.980	0.550	0.226	0.254	0.383	0.118	0.278	0.097	0.706	0.084	0.737	0.729	0.738	0.697	0.590	0.676	0.409	0.982
		N	631	631	631	631	631	631	631	631	509	509	509	509	509	509	509	509	122	122	122	122	122	122	122	122
Age (years)	≤55	ρ	−0.028	−0.091	0.042	−0.062	−0.002	−0.020	0.027	−0.009	−0.035	−0.074	0.002	−0.054	−0.006	0.007	−0.001	0.002	0.004	−0.267	0.246	−0.093	0.047	−0.290	0.396	−0.123
		P	0.710	0.232	0.581	0.420	0.975	0.798	0.720	0.907	0.674	0.376	0.981	0.519	0.944	0.929	0.987	0.982	0.986	0.178	0.217	0.643	0.816	0.142	**0.041**	0.540
		N	173	173	173	173	173	173	173	173	146	146	146	146	146	146	146	146	27	27	27	27	27	27	27	27
	>55	ρ	0.043	0.095	0.006	0.088	0.012	0.039	0.006	0.037	0.081	0.129	0.025	0.130	0.069	0.121	0.022	0.116	0.028	0.077	0.006	0.063	0.060	−0.101	0.133	−0.029
		P	0.361	**0.041**	0.901	0.060	0.795	0.402	0.903	0.433	0.123	**0.014**	0.633	**0.014**	0.192	**0.021**	0.671	**0.028**	0.788	0.459	0.950	0.547	0.561	0.331	0.200	0.782
		N	458	458	458	458	458	458	458	458	363	363	363	363	363	363	363	363	95	95	95	95	95	95	95	95
Sex	Male	ρ	0.017	−0.019	0.036	0.001	0.001	0.076	−0.032	0.052	0.043	0.029	0.025	0.043	0.040	0.174	−0.052	0.125	0.034	−0.105	0.142	−0.050	−0.041	−0.107	0.093	−0.092
		P	0.782	0.753	0.555	0.990	0.993	0.211	0.606	0.398	0.538	0.677	0.716	0.537	0.561	**0.011**	0.456	0.070	0.795	0.424	0.280	0.704	0.754	0.414	0.478	0.485
		N	270	270	270	270	270	270	270	270	210	210	210	210	210	210	210	210	60	60	60	60	60	60	60	60
	Female	ρ	0.034	0.077	0.018	0.073	0.003	−0.024	0.022	−0.008	0.059	0.069	0.044	0.086	0.057	0.006	0.063	0.044	0.022	0.163	−0.018	0.092	0.155	−0.024	0.122	0.054
		P	0.515	0.146	0.728	0.168	0.956	0.644	0.677	0.875	0.307	0.236	0.452	0.140	0.325	0.915	0.278	0.446	0.864	0.206	0.888	0.475	0.229	0.850	0.347	0.676
		N	361	361	361	361	361	361	361	361	299	299	299	299	299	299	299	299	62	62	62	62	62	62	62	62
Smoking	No	ρ	0.041	0.056	0.035	0.063	−0.002	−0.015	0.025	−0.004	0.068	0.076	0.049	0.094	0.040	0.044	0.035	0.057	0.032	0.055	0.035	0.048	0.027	−0.162	0.145	−0.096
		P	0.364	0.207	0.436	0.156	0.958	0.746	0.575	0.929	0.164	0.120	0.317	0.054	0.411	0.373	0.476	0.249	0.774	0.617	0.753	0.660	0.803	0.139	0.184	0.381
		N	502	502	502	502	502	502	502	502	417	417	417	417	417	417	417	417	85	85	85	85	85	85	85	85
	Yes	ρ	−0.043	−0.062	−0.010	−0.062	0.016	0.153	−0.100	0.132	−0.046	−0.056	−0.026	−0.081	0.056	0.194	−0.083	0.161	0.041	−0.025	0.052	0.009	0.100	0.260	−0.085	0.204
		P	0.627	0.484	0.907	0.483	0.853	0.084	0.259	0.135	0.661	0.597	0.804	0.443	0.594	0.065	0.429	0.126	0.810	0.883	0.760	0.956	0.554	0.120	0.618	0.226
		N	129	129	129	129	129	129	129	129	92	92	92	92	92	92	92	92	37	37	37	37	37	37	37	37
Drinking	No	ρ	0.004	0.055	−0.005	0.042	0.013	0.017	0.023	0.024	0.026	0.059	0.013	0.059	0.050	0.056	0.038	0.068	−0.046	0.110	−0.073	0.040	0.080	−0.043	0.130	0.016
		P	0.925	0.213	0.912	0.347	0.762	0.698	0.602	0.589	0.598	0.227	0.786	0.223	0.305	0.253	0.439	0.162	0.678	0.315	0.507	0.718	0.469	0.695	0.236	0.883
		N	508	508	508	508	508	508	508	508	423	423	423	423	423	423	423	423	85	85	85	85	85	85	85	85
	Yes	ρ	0.144	−0.006	0.147	0.087	−0.080	−0.001	−0.095	−0.036	0.175	0.056	0.132	0.135	−0.024	0.089	−0.075	0.033	0.205	−0.072	0.300	0.046	0.010	−0.006	0.013	0.015
		P	0.113	0.950	0.106	0.338	0.382	0.993	0.296	0.691	0.108	0.607	0.224	0.215	0.827	0.416	0.491	0.762	0.223	0.671	0.071	0.788	0.954	0.972	0.941	0.928
		N	123	123	123	123	123	123	123	123	86	86	86	86	86	86	86	86	37	37	37	37	37	37	37	37

[a] Spearman correlation test was performed. Abbreviations: w/o, without; AHD, antihypertensive drug; SBP, systolic blood pressure; DBP, diastolic blood pressure; PP, pulse pressure; MAP, mean arterial pressure.

Supplementary Table 9 **Correlation between *KLFs* mRNA expression and other quantitative traits**[a]

		KLF4	*KLF5*	hs-CRP	SBP	DBP	GLU	TC	TG	HDL-C
KLF5	*ρ*	0.354								
	P	**<0.001**								
	n	631								
hs-CRP	*ρ*	−0.121	−0.027							
	P	**0.041**	0.650							
	n	285	285							
SBP	*ρ*	0.029	0.005	0.039						
	P	0.470	0.905	0.513						
	n	631	631	285						
DBP	*ρ*	0.033	0.025	0.034	0.509					
	P	0.402	0.536	0.572	**<0.001**					
	n	631	631	285	631					
GLU	*ρ*	−0.063	−0.046	0.131	0.223	0.110				
	P	0.112	0.253	**0.027**	**<0.001**	**0.006**				
	n	631	631	285	631	631				
TC	*ρ*	−0.028	−0.006	−0.032	0.103	−0.031	0.177			
	P	0.480	0.871	0.587	**0.009**	0.439	**<0.001**			
	n	631	631	285	631	631	631			
TG	*ρ*	−0.009	−0.012	0.186	0.151	0.143	0.269	0.314		
	P	0.825	0.764	**0.002**	**<0.001**	**<0.001**	**<0.001**	**<0.001**		
	n	631	631	285	631	631	631	631		
HDL-C	*ρ*	−0.055	−0.084	−0.266	0.078	−0.148	0.048	0.376	−0.358	
	P	0.166	**0.034**	**<0.001**	0.051	**<0.001**	0.232	**<0.001**	**<0.001**	
	n	631	631	285	631	631	631	631	631	
LDL-C	*ρ*	0.002	0.048	0.052	0.073	0.013	0.156	0.885	0.214	0.160
	P	0.959	0.229	0.379	0.066	0.753	**<0.001**	**<0.001**	**<0.001**	**<0.001**
	n	631	631	285	631	631	631	631	631	631

[a]Spearman correlation test was performed. Abbreviations: hs-CRP, hypersensitive C-reactive protein; SBP, systolic blood pressure; DBP, diastolic blood pressure; GLU, glucose; TC, total cholesterol; TG, triglyceride; HDL-C, high density lipoprotein-cholesterol; LDL-C, low density lipoprotein-cholesterol.

Supplementary Table 10 **Sensitivity analyses between the polymorphisms of KLFs and the risk of hypertension in Yixing population**

Gene	SNP	Genotype	Groups		OR (95% CI); P-value			OR (95% CI); P-value[a]		
			Control (n)	HT (n)	Additive model	Dominant model	Recessive model	Additive model	Dominant model	Recessive model
KLF4	rs2236599	CC	997	966						
		CT	886	834						
		TT	189	189	0.998 (0.908–1.097); 0.973	0.982 (0.868–1.111); 0.775	1.046 (0.847–1.293); 0.676	1.010 (0.917–1.112); 0.845	0.995 (0.878–1.129); 0.942	1.064 (0.857–1.320); 0.574
			P_{HWE} = 0.696	P_{HWE} = 0.644						
	rs11841945	GG	1 273	1 202						
		GC	679	684						
		CC	120	103	1.011 (0.912–1.121); 0.834	1.043 (0.920–1.183); 0.511	0.889 (0.678–1.165); 0.393	1.009 (0.908–1.120); 0.871	1.041 (0.915–1.184); 0.542	0.884 (0.671–1.165); 0.380
			P_{HWE} = 0.021	P_{HWE} = 0.657						
KLF5	rs9573096	CC	975	875						
		CT	905	897						
		TT	192	217	1.116 (1.016–1.225); **0.022**	1.131 (1.000–1.280); **0.050**	1.199 (0.977–1.471); 0.082	1.118 (1.016–1.230); **0.023**	1.142 (1.007–1.296); **0.039**	1.179 (0.957–1.453); 0.122
			P_{HWE} = 0.385	P_{HWE} = 0.568						
	rs3812852	AA	1 805	1 733						
		AG	244	237						
		GG	23	19	0.988 (0.838–1.164); 0.885	0.999 (0.831–1.200); 0.988	0.860 (0.467–1.583); 0.627	0.973 (0.823–1.150); 0.747	0.986 (0.818–1.189); 0.883	0.798 (0.427–1.492); 0.480
			P_{HWE} <0.001	P_{HWE} <0.001						

[a]Adjusted for age, sex, smoking, drinking, diabetes, dyslipidemia, and body mass index.
The total number of participants from the Yixing cohort (including Yicheng) is 4 061, which is the sum of the relevant samples used in the study.
Abbreciations: WT, wild type; HT*, heterozygote type; MT, mutant type; OR, odds ratio; CI, confidence interval; HWE, Hardy-Weinberg equilibrium; HT, hypertension.

Supplementary Table 11 **Sensitivity analyses of *KLFs* with the incidence risk of hypertension by extracting subjects with a follow-up period of ⩾ one year**

Gene	SNP	Genotype	Total (*n*)	Incidence (*n*)	Person-years	Incidence density/ (10^4 person-years)	HR (95% CI); *P*-value			HR (95% CI); *P*-value[a]		
							Additive model	Dominant model	Recessive model	Additive model	Dominant model	Recessive model
KLF4	rs2236599	CC	982	293	9 738.52	300.87						
		CT	890	287	8 668.21	331.09						
		TT	197	63	1 920.78	327.99	1.070 (0.952–1.204); 0.255	1.103 (0.945–1.289); 0.214	1.055 (0.814–1.369); 0.685	1.078 (0.958–1.212); 0.212	1.116 (0.955–1.303); 0.168	1.058 (0.816–1.373); 0.670
	rs11841945	GG	1 287	396	12 687.40	312.12						
		GC	662	206	6 504.51	316.70						
		CC	120	41	1 135.60	361.04	1.042 (0.917–1.184); 0.527	1.027 (0.876–1.204); 0.739	1.166 (0.850–1.599); 0.342	1.034 (0.910–1.175); 0.608	1.019 (0.869–1.195); 0.817	1.147 (0.835–1.574); 0.397
KLF5	rs9573096	CC	968	281	9 668.51	290.63						
		CT	905	287	8 808.80	325.81						
		TT	196	75	1 850.20	405.36	1.159 (1.031–1.301); **0.013**	1.168 (0.999–1.365); **0.051**	1.308 (1.028–1.665); **0.029**	1.189 (1.059–1.336); **0.004**	1.207 (1.033–1.411); **0.018**	1.354 (1.063–1.723); **0.014**
	rs3812852	AA	1 798	560	17 664.54	317.02						
		AG	243	71	2 399.51	295.89						
		GG	28	12	263.46	455.48	1.025 (0.842–1.247); 0.807	0.989 (0.786–1.246); 0.927	1.366 (0.772–2.417); 0.284	1.049 (0.864–1.275); 0.628	1.034 (0.820–1.302); 0.779	1.258 (0.709–2.231); 0.433

[a]Adjusted for age, sex, smoking, drinking, diabetes, dyslipidemia, and BMI.
Bold fonts indicate *P*-values < 0.05.
Abbreviation: HR, hazards ratio.

Supplementary Table 12 **Sensitivity analyses of *KLFs* with the incidence risk of stroke by extracting subjects with a follow-up period of ⩾ one year**

Gene	SNP	Genotype	Total (*n*)	Incidence (*n*)	Person-years	Incidence density/ (10^4 person-years)	HR (95% CI); *P*-value			HR (95% CI); *P*-value[a]		
							Additive model	Dominant model	Recessive model	Additive model	Dominant model	Recessive model
KLF4	rs2236599	CC	958	128	10 808.80	118.42						
		CT	816	109	9 242.44	117.93						
		TT	191	26	2097.18	123.98	1.015 (0.844–1.221); 0.873	1.007 (0.791–1.282); 0.956	1.056 (0.704–1.583); 0.792	0.989 (0.822–1.190); 0.904	0.975 (0.765–1.242); 0.836	1.017 (0.678–1.525); 0.935
	rs11841945	GG	1 181	156	13 323.64	117.09						
		GC	685	88	7 752.37	113.51						
		CC	99	19	1 072.41	177.17	1.097 (0.897–1.342); 0.367	1.036 (0.810–1.325); 0.776	1.538 (0.965–2.454); 0.071	1.141 (0.931–1.398); 0.203	1.076 (0.841–1.378); 0.560	1.681 (1.052–2.687); **0.030**
KLF5	rs9573096	CC	901	126	10 096.34	124.80						
		CT	865	113	9 810.74	115.18						
		TT	199	24	2 241.34	107.08	0.924 (0.766–1.113); 0.405	0.909 (0.713–1.157); 0.438	0.892 (0.586–1.357); 0.593	0.917 (0.762–1.104); 0.361	0.919 (0.721–1.172); 0.498	0.830 (0.545–1.264); 0.385
	rs3812852	AA	1 705	222	19 260.16	115.26						
		AG	241	39	2 678.50	145.60						
		GG	19	2	209.75	95.35	1.172 (0.868–1.582); 0.299	1.235 (0.885–1.723); 0.214	0.797 (0.198–3.203); 0.749	1.203 (0.882–1.641); 0.243	1.218 (0.871–1.702); 0.249	1.343 (0.332–5.441); 0.680

[a]Adjusted for age, sex, smoking, drinking, diabetes, dyslipidemia, and BMI.
Bold fonts indicate *P*-values < 0.05.
Abbreviation: HR, hazards ratio.

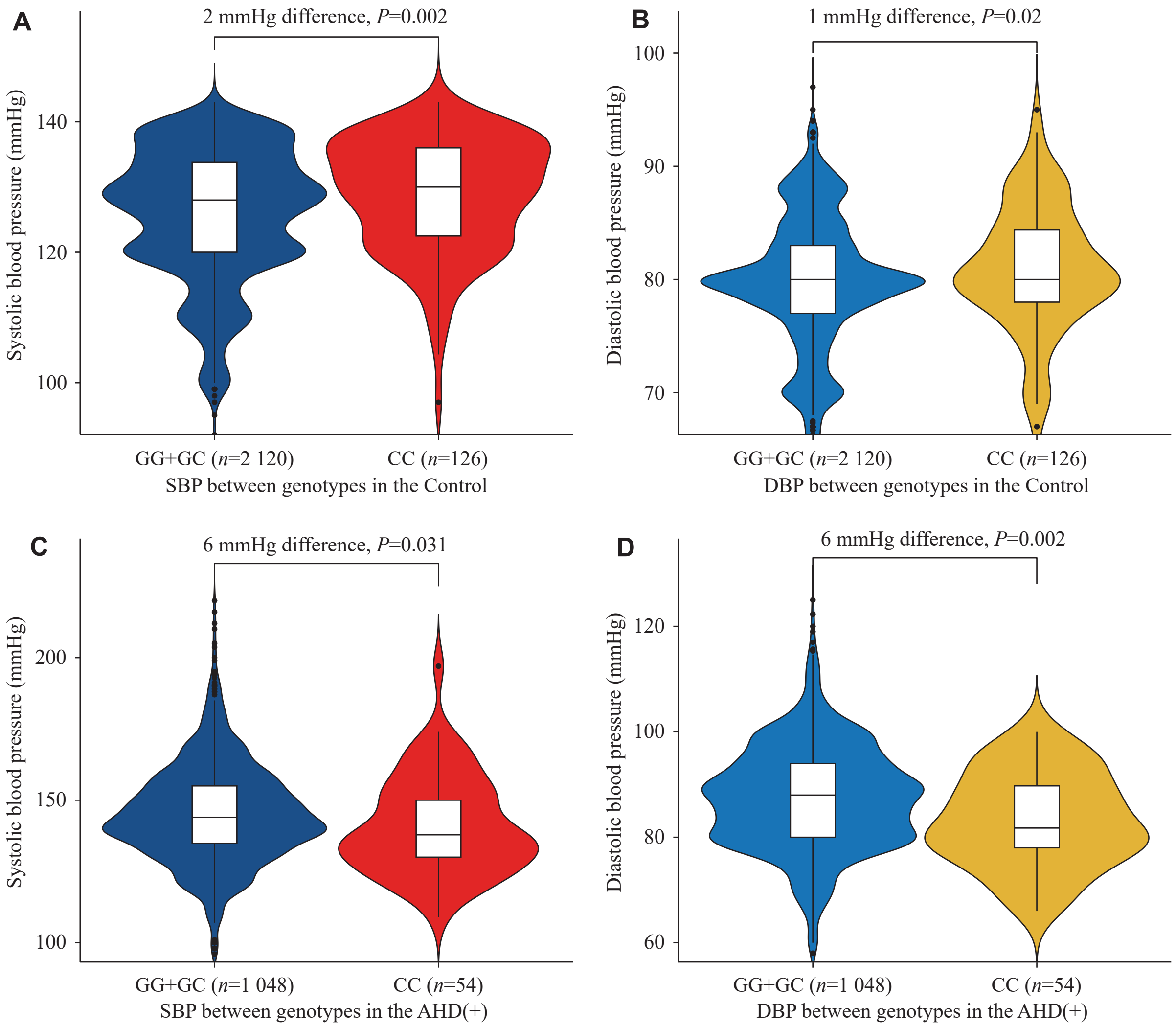

Supplementary Fig. 1 **Blood pressure between different genotypes of *KLF5* rs11841945.** A: The comparison of SBP between GG+GC and CC genotypes in the control group. B: The comparison of DBP between GG+GC and CC genotypes in the control group. C: The comparison of SBP between GG+GC and CC genotypes in the AHD(+) group. D: The comparison of DBP between GG+GC and CC genotypes in the AHD(+) group. Mann-Whitney *U* test was used to analyze between the groups. Abbreviations: SBP, systolic blood pressure; DBP, diastolic blood pressure.

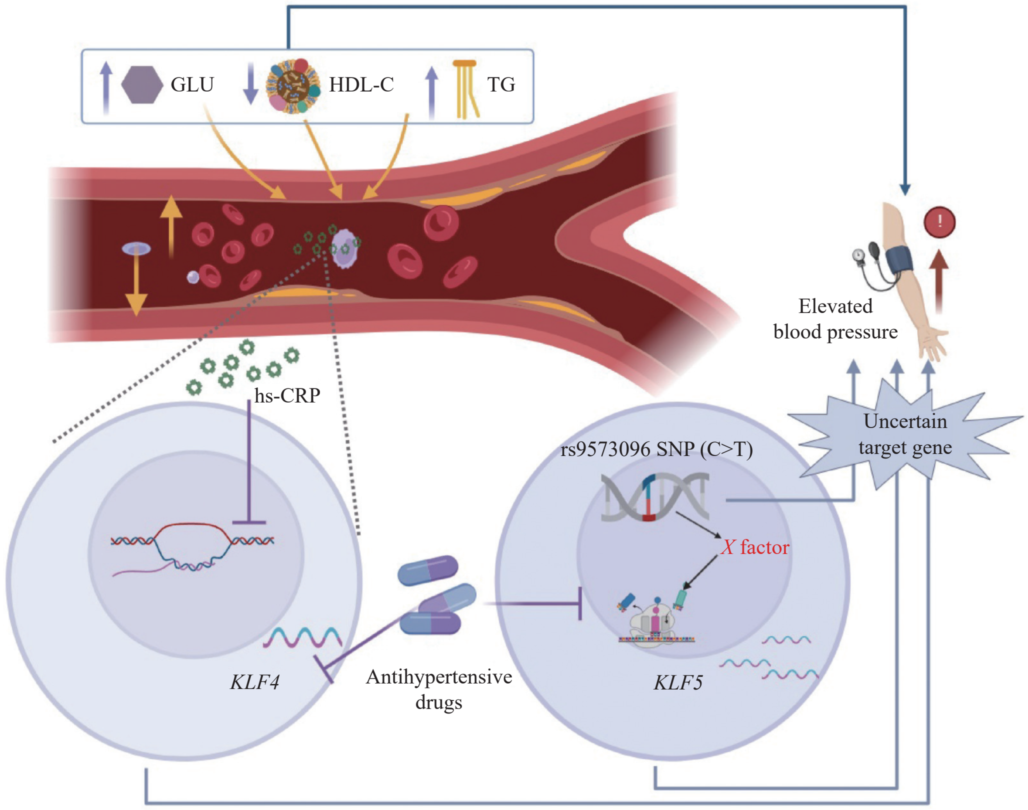

Supplementary Fig. 2 **Correlations among *KLF4* and *KLF5* mRNA expression levels, as well as hs-CRP and other quantitative traits.** Correlation analyses revealed positive correlations of hs-CRP levels with GLU (ρ = 0.131, P = 0.027) and TG (ρ = 0.186, P = 0.002), and a negative correlation with HDL-C (ρ = −0.266, P <0.001). There is no correlation between hs-CRP levels and blood pressure values (P > 0.05). Additionally, a negative correlation between *KLF4* mRNA expression and hs-CRP was observed (ρ = −0.121, P = 0.041). Glycemic and lipid indices also showed some correlation with blood pressure values. Abbreviations: GLU, glucose; HDL-C, high-density lipoprotein-cholesterol; TG, triglyceride; hs-CRP, hypersensitive C-reactive protein. (Created by BioRender.com).

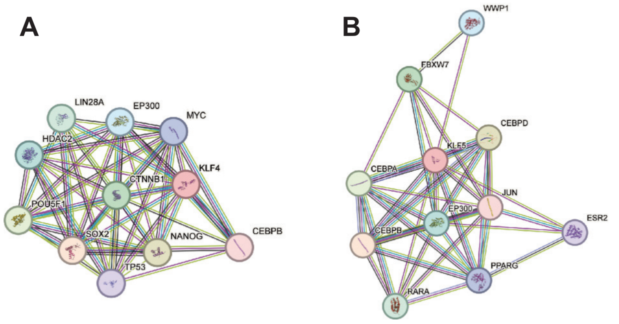

Supplementary Fig. 3 **Protein-protein interaction (PPI) networks of KLF4 and KLF5.** A: PPI networks of KLF4. B: PPI networks of KLF5.
